# Supplementary material for: Content-rich biological network constructed by mining PubMed abstracts
Source: BMC Bioinformatics. 2004 Oct 8;5:147. doi: 10.1186/1471-2105-5-147 (PMC528731; doi:10.1186/1471-2105-5-147)
Supplement: Additional File 2 — The original results of the above study (non-essential files are deleted to keep the file size under the limit set by BMC bioinformatics). [file 1471-2105-5-147-S2.bz2 › chilibotAdditionalFile2/dip30/209ID8846021E709/html/ITGA2_ITGB1.html]

 


 **ITGA2** and **ITGB1** 
  
Found 69 abstracts in PubMed, retrieved 30.  
 

 What does Google say? 
 PDF only 
| .edu only 

---

**Interactive relationship** (e.g. stimulation, inhibition, etc)

**Neutral relationship**- The human integrin very late antigen VLA 2 CD49b  [ **ITGA2** ]  CD29  [ **ITGB1** ]  mediates interactions with collagen and is the receptor for echovirus 1.  Ref: 9353313 J Biol Chem, 1997

**Non-interactive relationship** (e.g. studied together, co-existance, homology, etc.)

- CONCLUSIONS In vivo selection and flow cytometric analysis revealed that Lea, sLea, CD49b  [ **ITGA2** ] , CD49c, and CD29  [ **ITGB1** ]  appear to be involved in the increase of metastatic activity.  Ref: 9733622 J Surg Res, 1998
- In the original line KM12SM, sLea, E cadherin, CD49b  [ **ITGA2** ] , CD49c, or CD29  [ **ITGB1** ]  were positive in more than 40% of the cells.  Ref: 9733622 J Surg Res, 1998
- As model system, we used living human umbilical vein endothelial cells HUVEC adhering to laminin coated surfaces and investigated the distribution of the alpha2beta1 CD29  [ **ITGB1** ]  CD49b  [ **ITGA2** ]  integrin at the contact zone of these cells.  Ref: 12521136 Cell Commun Adhes< MedlineTA>Cell Commun Adhes, 1998
- Data obtained are presented in concurrence with Clusters of Thymic Epithelial Staining CTES classification for various monoclonal antibodies recognizing CD antigens CD1, CD1a, CD6, CD9, CD14, CD16, CD29  [ **ITGB1** ] , CD30, CD32, CD44, CD45RB, CD47, CD48, CD49a, CD49b  [ **ITGA2** ] , CD49c, CD49d, CD49e, CD49f, CD51, CD53, CD54, CD56, CD57, CD63, CD85, CD95, CD98, CD102, CD103, CD106, CD109, CD146, CD147, CD148, CD151, CD152, CD158a, CD158b, CD164, CD165, CD166 and for monoclonal antibodies 1B1 G7, A4, BD46, BLTZ, HP1C5, IND.64, M72, WU947 whose specifities are not yet defined.  Ref: 12725440 Ann Anat, 2003
- We found that T h 1 cells derived from ovalbumin OVA specific TCR transgenic DO11.10 mice showed significantly higher levels of VLA 2 CD49b  [ **ITGA2** ]  CD29  [ **ITGB1** ]  expression than T h 2 cells.  Ref: 12750354 Int Immunol, 2003
- OBJECTIVE We sought to determine whether expression of CD51 vitronectin and fibronectin receptor, CD54 intercellular adhesion molecule 1, HLA DR activation marker, CD29  [ **ITGB1** ]  beta1 integrin, CD49b  [ **ITGA2** ]  collagen receptor  [ **ITGA2** ] , and CD11b complement receptor exhibit a circadian rhythm in asthma.  Ref: 9768576 J Allergy Clin Immunol, 1998
- Flow cytometry of the cell surface antigen expression indicated that CD49b  [ **ITGA2** ] , CD29  [ **ITGB1** ] , carcinoembryonic antigen CEA, sialyl Lewis a sLea, and CD49c were positive in more than 70% of the cells.  Ref: 10853016 Int J Oncol, 2000
- Localization of integrin alpha2  [ **ITGA2** ]  and integrin beta1  [ **ITGB1** ]  in human stellate cells was examined by immunoelectron microscopy.  Ref: 9566533 Anat Rec, 1998
- Immunostaining was performed with a mouse monoclonal anti human integrin alpha2  [ **ITGA2** ]  or integrin beta1  [ **ITGB1** ]  antibody and goat anti mouse IgG coupled with 10 nm immunogold.  Ref: 9566533 Anat Rec, 1998
- Integrin alpha2  [ **ITGA2** ]  and integrin beta1  [ **ITGB1** ]  were detected on the surfaces of cell bodies, cellular processes, and microprojections.  Ref: 9566533 Anat Rec, 1998
- TEC were treated with TCDD at concentrations of 0.00.0..0 or 10.0 nM or with 100 nM PCB 126,, 5 pentachlorobiphenyl for 3 days, and were then analysed by flow cytometry for expression of surface antigens using monoclonal antibodies against Hassall s bodies TE 8, TE 16 or against surface structures such as CD29  [ **ITGB1** ] , CD49b  [ **ITGA2** ] , CD49e, CD49f, CD51, CD54, CD58, CD61 and CD106.  Ref: 12799776 Arch Toxicol, 2003
- This platelet aggregation activity was dose dependently inhibited by specific GPIbalpha antibodies, but not by those antibodies against platelet GPIa  [ **ITGA2** ] , GPIIa  [ **ITGB1** ] , GPIIb and GPIIIa.  Ref: 12782089 Toxicon< MedlineTA>Toxicon, 2003
- Anti CD9, CD26, CD29  [ **ITGB1** ] , CD31, CD36, CD44, CD49a, CD49b  [ **ITGA2** ] , CD49c, CD49d, CD49e, CD49f, CD54, CD55, CD56, CD62E &.  Ref: 10235320 J Pediatr Surg, 1999
- Other molecules expressed by the cultured stromal cells include CD29  [ **ITGB1** ] , CD49a, CD49b  [ **ITGA2** ] , CD49d, CD49f, CD51 61, CD54, CD55, CD56, CD58, CD59, CD61, CD71, CD79b, CD81, CD82, CD90, CD95, CD107a, CD130, and CD147.  Ref: 10951495 Prostate, 2000
- Flow cytometric analyses were carried out to detect expression of antigens Lewis a Lea, Lewis x Lex, sialyl Lewis a sLea, sialyl Lewis x sLex, E cadherin, CD44v6, integrin alpha2  [ **ITGA2** ]  CD49b  [ **ITGA2** ] , integrin alpha3 CD49c, integrin alpha4 CD49d, integrin alpha5 CD49e, and integrin beta1  [ **ITGB1** ]  CD29  [ **ITGB1** ] .  Ref: 9733622 J Surg Res, 1998
- We confirmed previous observations that human bronchial epithelial cells constitutively express CD29  [ **ITGB1** ] , CD44, CD49a, CD49b  [ **ITGA2** ] , CD49c, CD49d, CD49e, CD49f, CD51, CD54, CD61, and HLA class 1.  Ref: 9374108 Am J Respir Cell Mol Biol, 1997
- At 14 weeks of culture, more mature CHMCs expressed CD11b, CD11c, CD29  [ **ITGB1** ] , CD49b  [ **ITGA2** ] , CD49c, CD49d, CD49e, CD51, CD61, and CD54 and weakly expressed CD18 and CD11a.  Ref: 11174197 J Allergy Clin Immunol, 2001
- The expression of adhesion structures on mesothelial cells such as CD44, the beta integrin chain CD29  [ **ITGB1** ] , the beta 3 integrin chain CD61 and alpha chains CD49 alpha, CD49b  [ **ITGA2** ] , CD49c, CD49e, and CD51 is described.  Ref: 10649685 Adv Perit Dial, 1998
